# Pelagic–benthic resource polymorphism in *Schizopygopsis thermalis* Herzenstein 1891 (Pisces, Cyprinidae) in a headwater lake in the Salween River system on the Tibetan Plateau

**DOI:** 10.1002/ece3.6470

**Published:** 2020-07-08

**Authors:** Jialing Qiao, Jiaxin Hu, Qin Xia, Ren Zhu, Kang Chen, Jie Zhao, Yunzhi Yan, Ling Chu, Dekui He

**Affiliations:** ^1^ Provincial Key Laboratory of Biotic Environment and Ecological Safety in Anhui, and College of Life Sciences Anhui Normal University Wuhu China; ^2^ Institute of Hydrobiology Chinese Academy of Sciences Wuhan China

**Keywords:** diets, growth, morphological differentiation, resource polymorphism, *Schizopygopsis thermalis*, Tibetan lake

## Abstract

Resource polymorphism is a ubiquitous phenomenon in vertebrates and may represent a critical intermediate stage in speciation. Freshwater lakes in high‐altitude areas represent a natural system for understanding resource polymorphism in fishes benefiting from diverse lacustrine environments and species‐poor fish assemblages. We report resource polymorphism in a cyprinid fish, *Schizopygopsis thermalis*, in Lake Amdo Tsonak Co, a headwater lake in the upper Salween River system. Two discrete intraspecific morphs, planktivorous and benthivorous, were identified according to geometric morphometrics and traditional univariate linear measures. The planktivorous morph exhibits a longer head, longer upper and lower jaw, larger asymptotic standard length (*L_∞_*), lower growth rate (*k*), and higher growth performance index (*φ*) than the benthivorous morph. With respect to descriptive traits, the planktivorous morph possesses a large, terminal mouth and obvious mucus pores on the cheek and chin, while the benthivorous morph is characterized by a more inferior mouth with a sharpen horny edge on the lower jaw and unconspicuous mucus pores. The discrete pelagic–benthic resources and low interspecific competition in the lake system might drive the initial differentiation of the two morphs, and partial spatial reproductive isolation in breeding further maintains and reinforces the differences between them.

## Graphical abstract

I believe you will enjoy reviewing this work because our resource polymorphism in Schizopygopsis thermalis results present a good example to understand the important intermediate stages of ecological speciation.

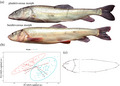

## 1 INTRODUCTION

Resource polymorphism is a phenomenon, whereby a single species exhibits two or more morphs (morphotypes) showing differences in morphology, behavior, coloration, or life history characteristics (e.g., growth characters) and potentially plays a critical role in population divergence and initial steps in speciation (Smith & Skúlason, 1996). Resource polymorphism has evolved in various vertebrates, including fishes, amphibians, and birds, but it has featured prominently in recently de‐glaciated systems, where resource‐based morphs can be often found at different stages of divergence within lakes, for example, three‐spined stickleback (*Gasterosteus*), whitefish (*Coregonus*), and charr (*Salvelinus*) species (Savvaitova, 1995; Skúlason et al., 2019). These taxa are of diverse phylogenetic origin and underwent rapid adaptive diversification. The polymorphism forms might be considered as an adaptation to habitat heterogeneity through the differentiation of feeding biology and habitat utilization. For example, two trophic morphs of three‐spined stickleback (*G. aculeatus*) coexist in several postglacial lakes, where one morph utilizes the littoral area and mainly feeds on benthic invertebrates while the other occupies the open‐water area and feeds on zooplankton (Schluter & Mcphail, 1992). The lake charr (*S. namaycush*) presented the lean and huronicus morphs adapting to shallow‐ and deep‐water environmental conditions (Chavarie et al., 2016). The morphological adaptations of such morphs appear to be similar among taxa; that is, the planktivorous morph often has longer and denser gill rakers, an elongated head and a longer lower jaw, while the benthivorous morph is characterized by shorter and sparser gill rakers, a blunt, round head, and a shorter lower jaw (Fraser, Adams, & Huntingford, 1998; Jonsson & Jonsson, 2001; Smith & Skúlason, 1996). In addition to the typical benthivorous–planktivorous pairs, three or more morphs also occur in some lake systems. European whitefish (*C. lavaretus*) has diverged into at least three sympatric morphs in the gill raker number and resulted in rapid adaptive radiation for the littoral, pelagic, and profundal lacustrine habitats in Fennoscandian postglacial lakes (Praebel et al., 2013). Three or more morphs of charrs, *S. namaycush* and *S. alpinus,* have co‐occurred in several postglacial lakes throughout the Holarctic (Hudson, Vonlanthen, & Seehausen, 2010; Jonsson & Jonsson, 2001; Muir, Hansen, Bronte, & Krueger, 2016). Although there were differences in the number of morphs among lakes, the observed morphological variations resulted from feeding strategies (e.g., benthivorous, planktivorous, invertivorous, or piscivorous) to adapting to the discrete food resources, which were depended on lacustrine environmental diversity and habitat preference.

Cyprinids, the largest family of vertebrates, also display diverse resource polymorphisms and with multiple morphs co‐occurring in a single freshwater system (Berrebi & Valiushok, 1998; de Graaf, Sibbing, & Osse, 2003). For example, the African barb (*Labeobarbus gananensis* complex) in the Genale River (Ethiopian highlands, East Africa) exhibits six forms differing in mouth morphology, gill rakers, diet, and gut length, and five of those were related to mouth morphology, which represents a typical form of adaptive radiation in response to different resources (Levin et al., 2019). The Schizothoracins (Cyprinidae), the largest and most diverse group of the Qinghai‐Tibetan Plateau ichthyofauna (He et al., 2020), also present substantial morphological specialization within species, in some cases, have evolved to forming new species by sympatric speciation within a single lake. For instance, four morphs of *Schizopygopsis stoliczkai* were found in Lake Yashilkul, Pamir (Savvaitova, Shanin, & Maksimov, 1987). Two sympatric species pairs, *Gymnocypris eckloni eckloni* and *G. e. scoliostomus* in Lake Sunmcuo (Zhao et al., 2009), and *G. chui* and *G. scleracanthus* in Lake Langcuo (Chen et al., 2020), have been demonstrated to exhibit a possible incipient sympatric adaptive ecological speciation by molecular phylogenetic relationships based on mitochondrial genes and genetic and expression variations of transcriptome, respectively.

Two morphs have found in *Schizopygopsis thermalis* Herzenstein 1891 (Cyprinidae: Schizothoracinae) in Lake Amdo Tsonak Co during field investigations in 2014–2018. The two morphs correspond to a resource axis in the lake: one form (planktivorous) predominately feeds on plankton and inhabits pelagic lake habitats, and the other form (benthivorous) mainly feeds on periphytic algae and dwells in the benthic zone and the tributaries (Nagchu River, Sang Chu) of the lake. The former morph possesses a normal lower jaw, a terminal mouth, while the latter morph is characterized by a shortened lower jaw, an inferior mouth with a sharpened horny edge on the lower jaw. However, the two morphs of *S. thermalis* have not been verified via morphological analysis. In addition, their biological characteristics (e.g., growth, feeding habit, and reproductive traits) are still unclear. To fill this knowledge gap and better understand the ecological mechanisms of polymorphism in *S. thermalis*, the specific objectives of this study were to (a) characterize the morphological variation of two distinct morphs coexisting in Lake Amdo Tsonak Co and quantitatively analyze their morphological characters by a combination of morphometric and traditional univariate linear measures; (b) specify the biological characters (e.g., growth, feeding habit, and reproductive traits) of two morphs of *S. thermalis*; and (c) elucidate the potential ecological mechanism of resource polymorphism in *S. thermali*s.

## 2 MATERIAL AND METHODS

### 2.1 Study area

Lake Amdo Tsonak Co (31.55–32.08°N, 91.25–91.33°E) is an oligotrophic freshwater lake of the headwater of Salween River (Nujiang) on the Tibetan Plateau, with a surface area of 182 km^2^, an elevation of 4,587 m above sea level, and a maximum depth of over 20 m. The annual mean air temperature is −3 to 0°C, the annual total rainfall is 350–420 mm, the conductivity ranges 569–633 μs/cm, the mean salinity is 0.29‰, the pH varied 8.61–9.00, the oxygen concentration ranged 5.63–7.60 mg/L, the phosphorus concentration varied 0.016–0.035 mg/L, the nitrogen concentration limited 0.18–0.62 mg/L, and water transparency extended 1.72–3.74 m.

**Figure 1:**
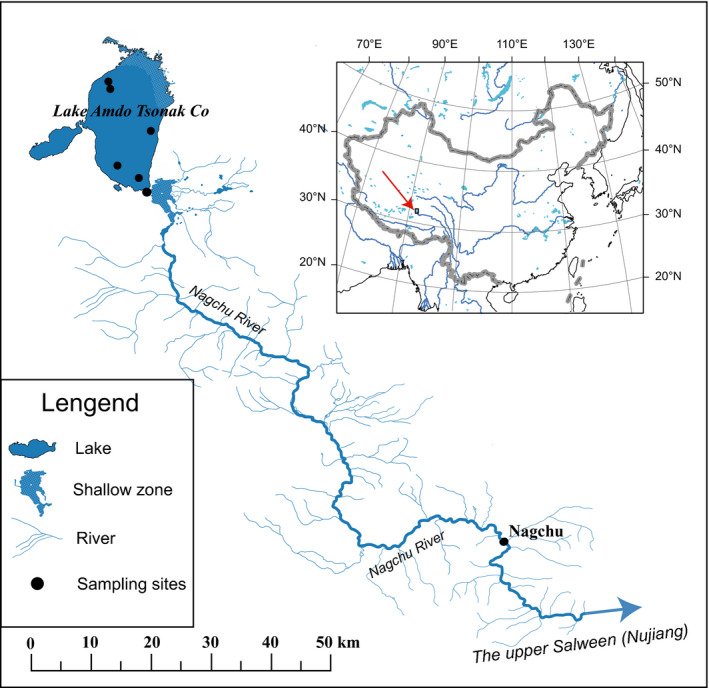
The sampling locations (black dots) in Lake Amdo Tsonak Co. Blue arrows represent the direction of the stream

The following zones are distinguished in Lake Amdo Tsonak Co: the shallow zone in the north and northeast of the Lake, where macrophytes predominate and benthic organisms (benthos) are abundant (Figure 1); the limnic zone (open‐water zone) in the west and south, where plankton dominates; and the deep‐water profundal zone, where stoneworts and zoobenthos are prevalent. The macrophytes mainly consist of *Myriophyllum*, *Potamogeton*, *Chara*, *Nitella*. The biomass of macrophytes varies from 100 to 10,000 mg/m^2^. Both phytoplankton and phytobenthos are represented by common diatoms, followed by green algae and blue‐green algae. The biomass of phytoplankton and phytobenthos varied 0.072 mg/L to 0.258 mg/L, 4.2 mg/L to‐13.2 mg/L during April and October, respectively. Zooplankton consists mainly of rotifers, cladoceran, copepod, and crustaceans, belonging to 46 species or genera. The biomass varied from 0.007 mg/L to 0.142 mg/L. Zoobenthos mainly consist of Oligochaeta (Tubificidae), Gastropoda (Lymnaeidae), and Arthropoda (*Gammarus* sp. and Chironomidae). Like phytobenthos, zoobenthos is most abundant in the shallow littoral zone in northeast of the Lake, and least so in the profundal in central and south of lake. The zoobenthos biomass varied from 9.40 g/m^2^ to 40.20 g/m^2^, and *Gammarus* sp. is the primary contributor. Only one highly specialized schizothoracin species, *S. thermalis*, and three Tibetan stone loaches (Nemacheilidae: *Triplophysa*) are distributed in this lake, and predatory fish are absent.

### 2.2 Field sampling

Fishes were collected from Lake Amdo Tsonak Co and its tributary (Nagchu River) (Figure 1) in May and September 2017 and April and July 2018 with gill nets (mesh size: 30 mm), cast nets, and hand nets. We labeled and photographed the left lateral side of each captured fish in the field. The standard length (SL, 0.1 mm), total length (TL, 0.1 mm), total weight (*W*), sex, and stage of maturity of each specimen were recorded in the field. Only adult individuals were considered in the subsequent analyses due to the less obvious characteristics of the juveniles. The gill rakes number on the outer row of the first gill‐arch was counted, and the maximum length of gill rakes was measured using vernier caliper (0.01 mm). We counted pharyngeal tooth row number and recorded the presence or absence of parasites. We also observed the development degree of mucus pores on cheek and chin. In addition, we also measured the width of the sharp horny edge of the lower jaw (Figure S1) and described the anterior border of lower jaw with or without sharp horny edge.

### 2.3 Morphological analysis

**Figure 2:**
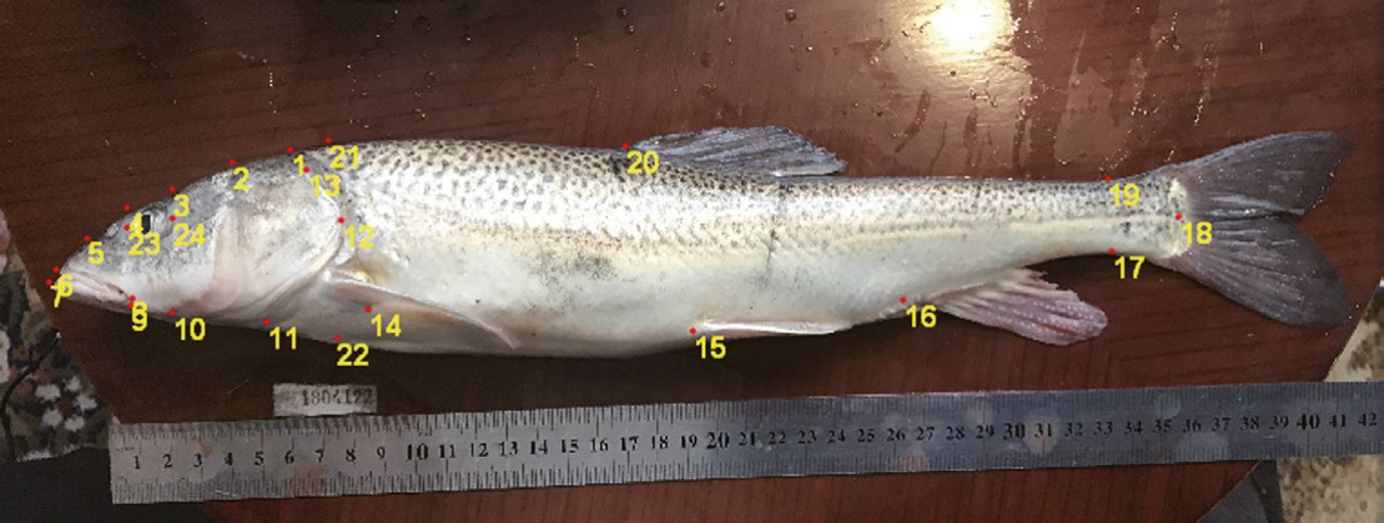
Landmarks used to measure the body shape of *Schizopygopsis thermalis* in Lake Amdo Tsonak Co: (1) posterior neurocranium above the tip of the opercle, (2) half the distance between the curve at points (1) and (3), (3)‐(10) posterior margin height of the eye, (4)‐(9) preocular margin height of the eye, (5) half the distance of the curve between (4) and (6), (6) and (7) anterior terminus of the upper and lower jaw, respectively, (8) posterior tip of the maxilla, (11) isthmus of the branchiostegal membrane, (12) endpoint of the opercle, (13) upper base of the operculum, (14) dorsal insertion location of the pectoral fin, (15) pelvic fin insertion location, (16) anterior insertion location of the anal fin, (17) to (19) minimum caudal peduncle depth, (18) hypural plate midpoint, (20) dorsal fin anterior insertion location, (21) to (22) show the head height, (23) preocular margin, and (24) posterior margin

With respect to the analysis of morphometric and linear traits, 154 specimens (*N* = 74 for planktivores; *N* = 80 for benthivores) were analyzed in this study. The left lateral side of the specimens was photographed using a Canon PowerShot G12 camera under natural light conditions with a ruler for scale. To obtain meaningful landmarks (homologous points), specimens were placed with the fins straightened and mouth closed in an orthogonal position. Specifically, the camera was fixed on a tripod with the lens parallel to the surface of the samples. Data on body shape and linear measurements for each fish were collected using digital landmarks on the photographs. A total of 24 landmarks were marked on each photograph (Figure 2) with tpsUtil and tpsDig2 (Rohlf, 2010). Body shapes were estimated by extracting the individual centroid size in MorphoJ 1.06 (Klingenberg, 2008). Simultaneously, new coordinates (*xy*) for each fish were extracted using generalized Procrustes superimposition for subsequent analysis (Markevich, Esin, & Anisimova, 2018). To increase the accuracy of body shape estimates, linear head traits (e.g., snout length, upper jaw length, lower jaw length, head length, head height and distance between the anterior termini of the upper, and lower jaws) were measured.

Allometry is common in fish, indicating that morphology and body size are typically related (Zelditch, Swiderski, Sheets, & Fink, 2004). Thus, a multivariate regression of body shape (Procrustes coordinates) on centroid size was used to correct for allometric effects, and regression residuals were used in geometric morphometric analyses (Elmer et al., 2014). Principal component analysis (PCA) was conducted via MorphoJ 1.06 to assess body shape variation (a geometric morphometric trait) among individuals without a priori grouping and to capture the maximum amount of variation with the smallest number of variables. The abbreviations and standardization of the linear head traits were as follows: HeadL = head length, SnoutL = snout length, Upper2 = upper jaw length, LowerL = lower jaw length and JawD = the distance between the anterior termini of the upper and lower jaws, which were normalized by *SL*, and HeadH = head height and UpperL1 = upper jaw length, which were standardized by head length and lower jaw length, respectively.

Morphs were initially identified with unweighted pair‐group method with arithmetic mean (UPGMA) cluster analysis using Past 3.2.6 (http://folk.uio.no/ohammer/past/) based on seven linear traits and scores from the first two principal components (PCs) of body shape by geometric morphometric analysis (Chavarie, Howland, & Tonn, 2013). To quantify the importance of each variable for the ordination axes, PCA of linear traits was then conducted. This procedure was performed with the ggbiplot package in R (version 0.55; Vu, 2011). Discriminant function analysis (DFA) and posterior jackknife cross‐validation were performed with SPSS 22.0 for morphs defined by cluster analysis to determine whether the morphs were significantly different. The efficacy of the DFA was evaluated with Wilks’ λ, which varies between 0 and 1, with zero indicating perfect identification. Finally, we performed multivariate analysis of variance (MANOVA) with Tukey's honestly significant difference (HSD) post hoc comparisons (SPSS 22.0) on body shape PCs and linear traits between morphs to test the validity of the discriminant analysis results.

Morphological difference between the sexes was estimated by implementing *t* tests of body shape PCs and linear traits. To visualize body shape differences between morphs, we reconstructed body shapes using landmark coordinates based on DFA of geometric morphometric data (Procrustes coordinates) (Elmer et al., 2014; Jakubavičiūtė, De Blick, Dainys, Ložys, & Olsson, 2018). For countable and measurable variables (e.g., gill raker number and row number of pharyngeal tooth; gill raker length), the Kolmogorov–Smirnov and Levene's tests were performed to estimate whether the data were normally distributed and homogenous. Analysis of variance (ANOVA) was conducted for variables that were normally distributed with variance homogeneity, and nonparametric tests were implemented for variables which were after logx transformed that still showed a non‐normal distribution or heteroscedasticity. To test whether the presence or absence of parasites affected body shape, analysis of covariance (ANCOVA) was performed using parasites as a covariate, morph as a factor and linear traits and PCs as dependent variables.

### 2.4 Diet analysis

To evaluate the feeding habits of the fish, we inspected the gut contents within the anterior intestine. In total, 25 individuals of the planktivorous morph and 30 individuals of the benthivorous morph were dissected under a dissecting stereomicroscope and the gelatinous substances of all individuals were excluded, such as gastric juices. Prey items were identified to the phylum, class, order, family, or genus level. Then, we divided all prey items into six categories: zooplankton, small fishes (including fishes and their remnants), hydrophilic insects, periphytic algae, zoobenthos, and others (hydrophyte debris, organic debris, and small grains of sand). First, the diet compositions were estimated by occurrence rate and wet weight percentage:

$$F\%=\left(N_{i}/N_{\text{total}}\right)\times 100\%$$

*F* % is the percentage of occurrence of prey *i*, *N_i_* is the frequency of occurrence of prey *i,* and *N_total_* represents the total number of gut samples with food:

$$W\%=\left(W_{c}/W_{\text{total}}\right)\times 100\%$$

*W %* is the wet weight percentage of prey category *c* (one of the six food categories), *W_c_* represents the wet weight of prey category *c*, and *W_total_* represents the total weight of all prey items in each sample. Schoener's index (*D_xy_*) (Schoener, 1970) of proportional diet overlap was calculated and used to evaluate the difference in food composition between the two morphs.

$$C_{\mathrm{xy}}=1-0.5\left\{\sum \left|P_{\mathrm{xc}}-P_{\mathrm{yc}}\right|\right\}$$

*C_xy_* represents the diet overlap index between the two forms (*x* and *y*). *P_xc_* and *P_yc_* represent the shared food category *c* of form *x* and *y* (*W %*), respectively. Values range between 0 (no diet overlap) and 1 (complete diet overlap), and values >0.6 generally indicate biologically significant overlap (Wallace, 1981). Finally, the nonparametric Kruskal–Wallis test was implemented to estimate the food composition (*W %*) difference between two morphs.

### 2.5 Growth analysis

In total, 140 specimens (lapillus otoliths) were used to estimating the growth characteristics of the two morphs (planktivorous, *N* = 66; benthivorous, *N* = 74). Preparation of otolith sections and age determination was performed by an experienced worker. Each otolith was interpreted three times, and otoliths without at least 2 identical interpretations were excluded from the analysis. Photographs of all otolith sections were captured using MicroPublisher (5.0 RTV) under a light microscope (BH2; Olympus Optical). Otolith radius and ring diameter were measured with Autook (Image Analyser 2.0).

The relationship of *SL* with otolith radius was described by Frase–Lee regression, and the back‐calculated *SL* of all ages was obtained using the modified Frase–Lee function (Johnson & Noltie, 1997):

$$\log_{e}L_{i}=a+\left(\log_{e}L_{c}-a\right)\left(\log_{e}O_{i}/\log_{e}O_{c}\right).$$

where *L_c_* is the *SL* of the specimens, *a* is the intercept of the *SL*‐otolith radius regression, *O_c_* is the radius of the otolith at capture, *O_i_* is the radius of the otolith at age *i* and *L_i_* is the back‐calculated *SL* at age *i*.

Back‐calculated *SL* was used to fit von Bertalanffy growth function (VBGF) (von Bertalanffy, 1938) and obtain the growth parameters of each morph:

$$L_{t}=L_{\infty }\left(1-\exp^{\left(-k\times (t-t0)\right)}\right).$$

where *L_t_* is the back‐calculated *SL* at age *t*, *L_∞_* is the asymptotic *SL*, *k* is the growth coefficient, *t* is age, and *t_0_* is the theoretical age at zero length.

To compare growth parameters, the growth performance index (*φ*) was calculated according to the equation of Munro and Pauly (1983):

$$\varphi =\log_{10}K+2\log_{10}L_{\infty }$$

where *K* is the growth coefficient and *L_∞_* is the asymptotic *SL*.

Because of the nonlinear formulation of the VBGFs, a general linear model could not be used for ANCOVA. Instead, an analysis of residuals of the sum of squares (ARSS) was performed to compare the VBGFs between sexes and morphs (Chen, Jackson, & Harvey, 1992), and the degree of fit was denoted by the correlation coefficient and coefficient of determination (*R^2^*).

### 2.6 Genetic analysis

Fin clip was collected from each specimen and preserved in 95% ethanol. Total genomic DNA was isolated using a standard phenol–chloroform method. Two mitochondrial gene sequences, the cytochrome *b* gene (Cyt *b*, 1,140 bp) and the control region (D‐loop, 839 bp), were amplified for planktivorous (*N* = 36) and benthivorous (*N* = 33) morphs, respectively. Detailed primer information is given in Table S1. The sequences of two genes were amplified and sequencing adopted from He and Chen (2006) and Liang, He, Jia, Sun, and Chen (2017).

The sequences were visually checked in BioEdit 7.0 (Hall, 1999) and aligned with ClustalX (Larkin et al., 2007). Five species of the genus *Schizopygopsis* (*S. younghusbandi*, *S. pylzovi*, *S. stoliczkai*, *S. bangongensis*, and *S. malacanthus*) and the closely related species *Herzensteinia microcephalus* were analyzed together. Two species of genus *Gymnocypris*, *G. eckloni,* and *G. przewalskii* were designated as outgroups. Phylogenetic relationships were constructed under maximum likelihood (ML) and Bayesian inference (BI) in CIPRES (Miller, Pfeiffer, & Schwartz, 2010) using a concatenated sequence of Cyt *b* and the D‐loop, respectively. Analysis of molecular variance (AMOVA, Excoffier, Smouse, & Quattro, 1992) was performed using Arlequin 3.5 (Excoffier & Lischer, 2010) to estimate the genetic differences among groups defined a priori morphs.

## 3 RESULTS

### 3.1 Morphological analysis

**Figure 3:**
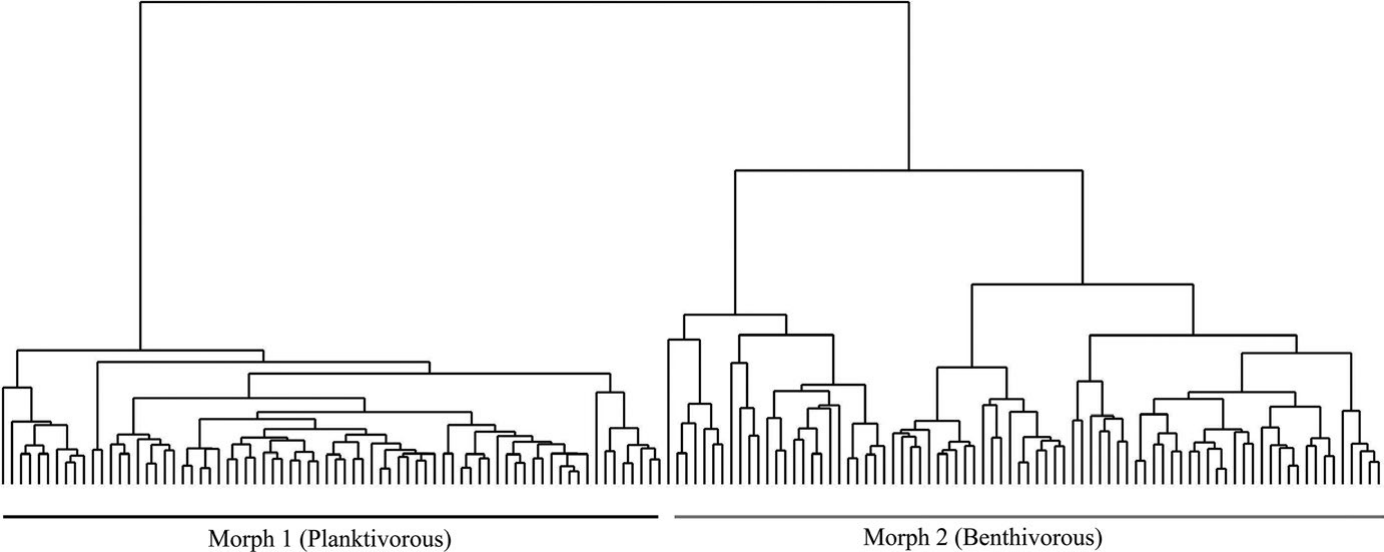
The dendrogram resulting from UPGMA cluster analysis of 154 *Schizopygopsis thermalis* from Lake Amdo Tsonak Co and its tributary, the Nagchu River. Two major morphs of *S. thermalis* were identified based on linear traits and PC scores of body shape measurements

**Figure 4:**
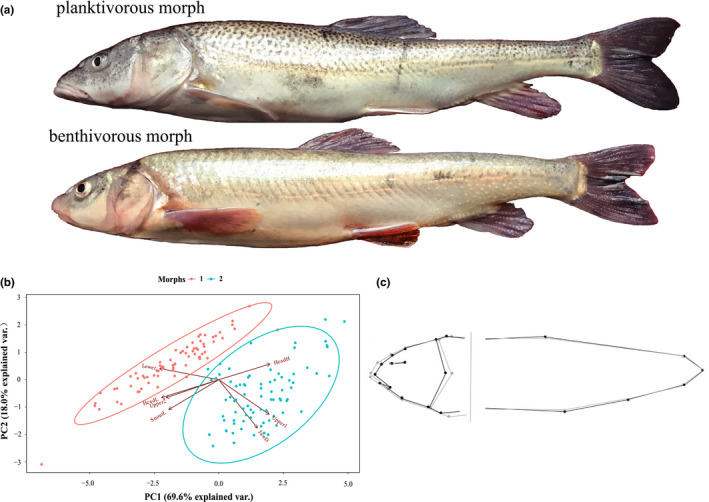
The PCA ordinations of linear traits of *Schizopygopsis thermalis* and the reconstructed average body shape of each morph. (a) The two morphs of *S. thermalis*, with the upper fish representing the planktivorous morph and the lower fish representing the benthivorous morph. (b) PC1 and PC2 explained 69.6% and 18.0% of the variation, respectively. The angles and lengths of the arrows represent the direction and strength of the relationships between variables and the principal components, respectively. Morphs were identified by UPGMA cluster analysis and delimited by 95% probability ellipses (*N* = 154), and the abbreviations of linear traits are explained in Table 1. (c) The average body shape difference between the two morphs based on DFA with only geometric morphometric data (Procrustes coordinates). The gray outline represents the planktivorous morph, while the black outline represents the benthivorous morph

Across all samples, two morphs were identified with UPGMA cluster analysis on the basis of body shape (Figure 3), referred to here as morph 1 (planktivorous, *N* = 74) and morph 2 (benthivorous, *N* = 80). DFA showed that the two morphs differed significantly in terms of body shape (Wilks’ λ = 0.13, *N* = 154, *p* < .001). A posteriori jackknife cross‐validation showed high success, with 98.6 and 98.8% of the samples being correctly allocated to the planktivorous and benthivorous morphs, respectively. According to the MANOVA results (Table 1), seven linear traits and two body shape PCs were effective variables for the discriminant analysis. Based on the PCA and reconstructed body shape results (Figure 4), the samples of the planktivorous morph had more terminal mouth, much longer and robust head shape, and longer upper jaw, lower jaw and snout length than those of the benthivorous morph. There was no significant difference in body shape PCs and linear traits between the sexes (*t*‐test, *df* = 152, all *p* > .05, males: females = 1:1.8). With regard to parasites, only cestodes and *Caenorhabditis elegans* were identified in a small number of samples (planktivorous, *N* = 3; benthivorous, *N* = 9). Parasitism also did not affect the morphology of the specimens (ANCOVA; *p* = 1.00, >.05).

**Table 1:** Linear trait means ± *SE*s based on traditional morphological measurements for two morphs of *Schizopygopsis thermalis* from Lake Amdo Tsonak Co. The linear trait means were normalized by *SL*, except for those of HeadH and UpperL1

Traits	Linear trait means ± *SE*s (Tukey HSD results)		MANOVA *p*‐value
	Planktivorous morph	Benthivorous morph	
HeadH	0.77 ± 0.01	0.86 ± 0.01	<.001
UpperL1	1.06 ± 0.01	1.72 ± 0.03	<.001
HeadL	0.23 ± 0.00	0.19 ± 0.00	<.001
SnoutL	0.06 ± 0.00	0.05 ± 0.00	<.001
Upper2	0.06 ± 0.00	0.05 ± 0.00	<.001
LowerL	0.05 ± 0.00	0.03 ± 0.00	<.001
JawD	0.01 ± 0.00	0.03 ± 0.00	<.001
Body shape PC1	_______	_______	<.001
Body shape PC2	_______	_______	.002

Note

The linear variable abbreviations in this table are as follows: HeadL = head length, SnoutL = snout length, Upper2 = upper jaw length, LowerL = lower jaw length and JawD = the distance between the anterior termini of the upper and lower jaws, which were normalized by *SL*, and HeadH = head height and UpperL1 = upper jaw length, which were standardized by head length and lower jaw length, respectively. The results of MANOVA comparing the linear traits and PC scores between the groups are also presented. Normalization could not be performed for PC scores.

With respect to descriptive traits, the two morphs significantly differed in head trophic traits. Planktivorous individuals possessed a terminal mouth with a slightly horny edge (width of horny edge: <0.02 cm) or lacked a horny edge on the anterior border of lower jaw, and both sides of cheek and chin present one line obvious mucus pores. The benthivorous individuals were characterized by a sub‐inferior mouth with sharpen horny edge on the lower jaw (width of horny edge: 0.14–0.42 cm) and the lack of mucus pores or only small mucus pores on the cheek and chin.

Although pharyngeal teeth in one or two rows were observed in both morphs (Figure S2), the percentage of individuals with a single row of pharyngeal teeth was higher in the benthivorous (30.0%) than planktivorous morph (10.8%). In contrast to most resource polymorphisms in postglacial lakes where gill rakers of morphs represent a typical trophic adaptive trait, there was no statistically significant differences in numbers and lengths of gill rakers between morphs (*t* test; all *p* > .05).

### 3.2 Diets

**Figure 5:**
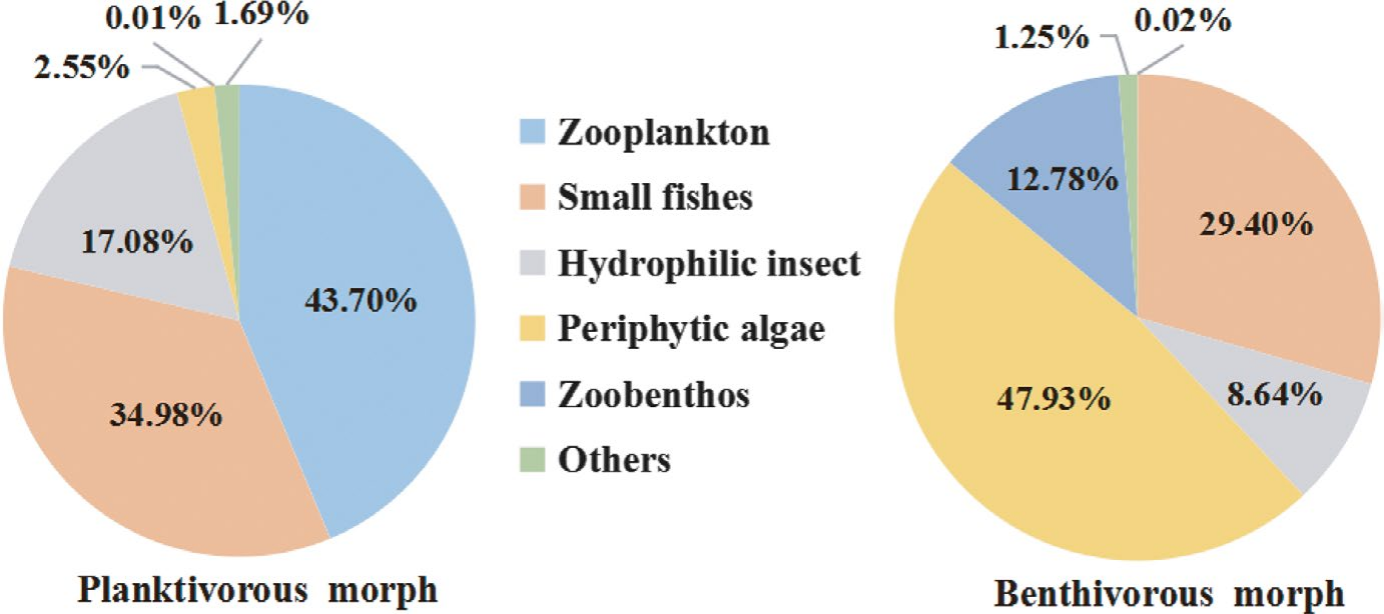
Pie chart of food composition for the two morphs. Note: Others include hydrophyte and organic debris

Diet segregation was clear in the two morphs of *S. thermalis* showing low dietary overlap (*C_xy_* = 0.42). The wet gut content weight of the planktivorous morph mainly consisted of zooplankton (43.7%), small fishes (34.98%), and hydrophilic insects (17.08%), while the benthivorous morph ingested a larger proportion of periphytic algae (47.93%) and zoobenthos (12.78%) (Figure 5). The nonparametric Kruskal–Wallis test showed that the wet percentage of food composition of periphytic algae (χ^2^: 40.30, *p* < .001), zooplankton (χ^2^: 46.28, *p* < .001), zoobenthos (χ^2^: 43.34, *p* < .001), hydrophilic insects (χ^2^: 25.58, *p* < .001), small fishes (χ^2^: 15.91, *p* < .001) differed significantly between the two morphs. The detailed dietary data are shown in Table S1.

### 3.3 Growth

Back calculation of otolith length was performed for males, females, and both sexes of each morph. There were no significant differences in the relationship of *SL* with otolith radius (ANCOVA after log transformation) between males and females of the planktivorous morph (*N* = 66, males: females: 1:1.87, *F* = 0.382, *p* = .539 > .05) or benthivorous morph (*N* = 74, males: females: 1:1.96, *F* = 3.297, *p* = .073 > .05). Therefore, the back‐calculated *SLs* of all ages were obtained using a modified Frase–Lee function for both sexes (males and females combined) of each morph, as follows:

planktivorous morph,

$$\log_{e}L_{i}=5.0789+\left(\log_{e}L_{c}-5.0789\right)\;\left(\log_{e}O_{i}/\log_{e}O_{c}\right)\;\left(R^{2}=\;0.9241\right)$$

benthivorous morph,

$$\log_{e}L_{i}=5.1594+\left(\log_{e}L_{c}-5.1594\right)\;\left(\log_{e}O_{i}/\log_{e}O_{c}\right)\;\left(R^{2}=0.8436\right)$$

**Figure 6:**
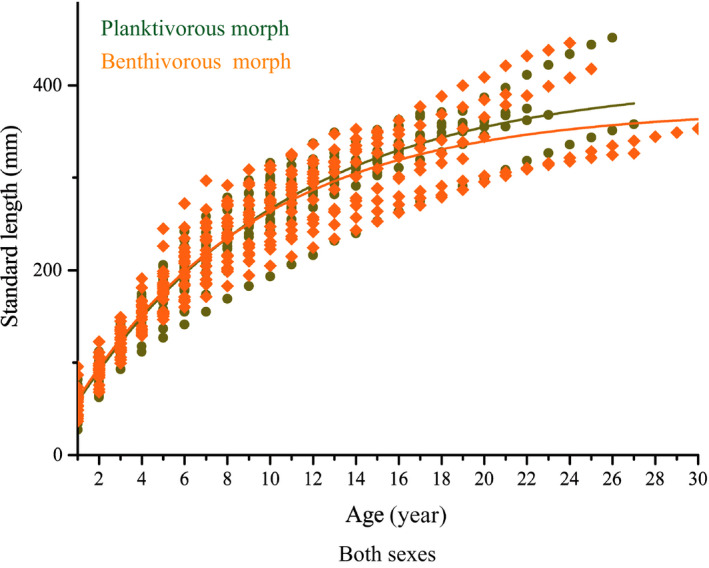
Relationship between age and the back‐calculated standard length for both sexes of the planktivorous morph and benthivorous morph

The back‐calculated *SL* of the samples (https://doi.org/10.5061/dryad.nvx0k6dnt) was used to fit a VBGF for females, males (Figure S3), and both sexes (Figure 6) of each morph, respectively.

For the planktivorous morph, we found that the females exhibited a larger asymptotic SL (*L_∞_* = 408.23), lower growth rate (*k* = 0.104), and higher growth performance index (*φ = *4.24) than males (*L_∞_* = 372.22, *k* = 0.12 and *φ = *4.22). However, for the benthivorous morph, the males presented a larger asymptotic SL (*L_∞_* = 371.88), higher growth rate (*k* = 0.139) and higher growth performance index (*φ = *4.28) than did females (*L_∞_* = 371.23, *k* = 0.12 and *φ = *4.22). According to the VBGF test performed via ARSS, we found a significant difference between males and females for each morph (planktivorous morph: *F*_0.01(65,64)_ < 4.45, *p* < .01; benthivorous morph: *F*_0.01(73,72)_ < 681.74, *p* < .01). With respect to the comparison of single gender’ growth characteristics between the two morphs, females of the planktivorous morph showed a larger asymptotic SL (*L_∞_* = 408.23), lower growth rate (*k* = 0.104), and higher growth performance index (*φ = *4.24) than those of the benthivorous morph (*L_∞_* = 371.23, *k* = 0.12 and *φ = *4.22). Males of the planktivorous morph exhibited a larger asymptotic SL (*L_∞_* = 372.22), lower growth rate (*k* = 0.12), and lower growth performance index (*φ = *4.22) than those of the benthivorous morph (*L_∞_* = 371.88, *k* = 0.139 and *φ = *4.28). The ARSS indicated that the VBGFs of females (*F*_0.01(91,90)_ < 115.86, *p* < .01) and males (*F*_0.01(47,46)_ < 47.82, *p* < .01) of the two morphs were significantly different, respectively. Further comparison about growth characteristics of both sexes, we found the individuals of planktivorous morph exhibited a larger asymptotic SL (*L_∞_* = 405.14), lower growth rate (*k* = 0.102), and higher growth performance index (*φ = *4.22) than individuals of the benthivorous morph (*L_∞_* = 374.22, *k* = 0.116 and *φ = *4.21). The significantly different VBGFs between morphs were supported by the ARSS results (*F*_0.05(139,138)_ = 1.64, *p* < .05). The detailed results are shown in Tables 2 and 3.

**Table 2:** The growth functions, parameters, and the growth performance index of two morphs

	Categories	Samples	*L*_max_	*K*	*t*_0_	*φ*	VB equation	*R* ^2^
Planktivorous morph	Both sexes	66	405.14	0.102	−0.507	4.22	$L=405.14(1-e^{\left(-0.102(t+0.575)\right)})$	0.932
	Female	43	408.23	0.104	−0.49	4.24	$L=408.23(1-e^{\left(-0.104*(t+0.49)\right)})$	0.918
	Male	23	372.22	0.12	−0.435	4.22	$L=372.22(1-e^{\left(-0.12*(t+0.435)\right)})$	0.947
Benthivorous morph	Both sexes	74	374.22	0.116	−0.511	4.21	$L=374.2(1-e^{\left(-0.116*(t+0.511)\right)})$	0.89
	Female	49	371.23	0.12	−0.331	4.22	$L=371.23(1-e^{\left(-0.12*(t+0.331)\right)})$	0.864
	Male	25	371.88	0.139	−0.019	4.28	$L=371.88(1-e^{\left(-0.139*(t+0.019)\right)})$	0.905

**Table 3:** The different kinds of von Bertalanffy growth functions compared by means of RSS

Categories	Comparison between	RSSp	DF	RSSs	DF	*F*	*p*
Planktivorous morph	Sexes	146,919.63	65	137,368.26	64	4.45	<.01
Benthivorous morph	Sexes	229,856.64	73	21,956.85	72	681.74	<.01
*Schizopygopsis thermalis*	Morphs	381,258.76	139	376,776.27	138	1.64	<.05
Female samples	Morphs	283,543.67	91	123,961.93	90	115.86	<.01
Male samples	Morphs	72,128.12	47	35,363.18	46	47.82	<.01

### 3.4 Phylogenetic analyses

**Figure 7:**
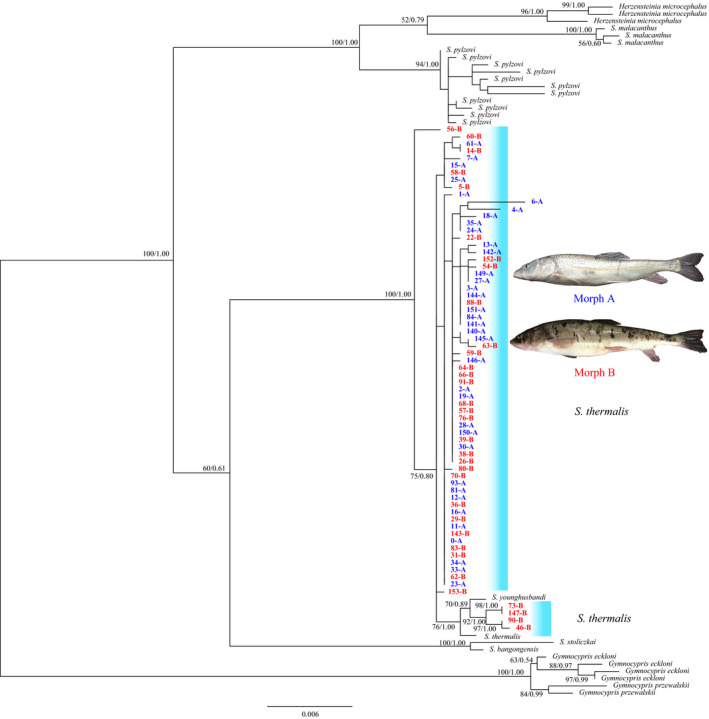
ML tree recovered from the combined Cyt *b* and D‐loop sequence data of *Schizopygopsis thermalis* and other closely related taxa assessed in the present study. The numbers on the branches correspond to bootstrap support of ≥60% obtained in the Bayesian and ML analyses

The ML and BI analysis produced similar topologies, using the concatenated sequence data (Cyt *b* + D‐Loop). With respect to outgroups and other closely related taxa, phylogenetic analyses showed that the individuals of the two morphs were genetically mixed and did not support the monophyly of two forms or *S. thermalis* (bootstrap support = 100; Bayesian posterior probability = 1.00), revealing by four individuals clustered with *S. younghusbandi* form the Yarlung Tsangpo Rivers (Figure 7). Similar results have been revealed on an extensive range phylogeographic analysis (He et al., 2016).

AMOVA testing indicated that the majority of genetic variation (97.01%) was from within morphs; only a small amount of the total genetic variation (2.99%) was attributed to variation between the two morphs. The genetic differentiation of two morphs was weak, but significant (*Ф*_ST_ = 0.0299, *p* < .05). However, no significant difference in two morphs was observed (*Ф*_ST_ = 0.0048, *p* = .2688) when four individuals clustered with *S*. *younghusbandi* were excluded from *S. thermalis*.

## 4 DISCUSSION

Resource polymorphism is a ubiquitous phenomenon in vertebrates and may represent a critical intermediate stage in speciation. To utilize the available discrete food resources in environments, species often display morphological divergence with a certain degree of polymorphism (Jonsson & Jonsson, 2001; Smith & Skúlason, 1996). In this study, we demonstrated the existence of two sympatric forms (planktivorous and benthivorous) in *S. thermalis* in Lake Amdo Tsonak Co by morphological and ecological analyses.

### 4.1 Morphological analysis

Our results showed that planktivorous and benthivorous morphs were clearly different in head shape. Head shape features are associated with the foraging strategy and trophic adaptation of species. The planktivorous morph has a large, terminal mouth, longer snout, upper and lower jaw, long head, even with a projecting lower jaw. These traits might contribute to predation on zooplankton and small fishes in open waters. In contrast, the benthivorous morph shows a more inferior mouth, a shorter snout and upper and lower jaw, and a blunt and short head, which might be conducive to predating on the benthic organisms living in the bottom sediments and organic detritus (Blackie, Weese, & Noakes, 2003; Levin et al., 2019; Markevich et al., 2018).

Two morphs also display obvious differences in the prehensile apparatus of lower jaw (e.g., the horny edges and mucus pores). The planktivorous morph presents a slight horny edge or absent a sharpened horny edge on the anterior border of the lower jaw and presents obvious mucus pores on the cheek and chin. In contrast, the benthivorous morph has a tendency toward a herbivorous (plant‐based) habit and has possessed a horny cutting edge on the lower jaw, and unconspicuous mucus pores, which facilitates scraping periphytic algae adhering to the bottom (Ji, 2008; Liang, 2017). The obvious mucus pores and canals on the cheek and chin are unique sensory organ in schizothoracins and only present species with a terminal mouth and without barbel (*Gymnocypris* and *Oxygymnocypris*). This curious feature may result from an apparent compensatory relationship between vision and taste, and in relation to the lateral line system or taste buds, but the function of which is not yet clearly understood. In cyprinids, taste buds may be useful for ensuring full utilization of the gustatory ability of the fish, and carnivorous fishes are endowed with taste buds (Gomahr, Palzenberger, & Kotrschal, 1992).

The number of pharyngeal tooth rows was also found to significantly differ between the two morphs, indicating that a larger proportion of benthivores than of planktivores had one row of such teeth. Pharyngeal teeth are associated with their feeding functions (e.g., grasping, holding of the prey, crushing, and grinding of various food items). Eastman (1971, 1977) indicated that pharyngeal teeth are important feeding organs used by fish to crush food and are present in all cyprinids (and other families). The difference in the number of pharyngeal tooth rows between morphs could result from functional responses to increased crushing force during mastication as the “tooth‐food‐chewing pad complex” is working (Smits, Witte, & Povel, 1996; Uzar, Andrzejewski, & Kozak, 2019).

In contrast to the traits divergence of morphs in European whitefish and three‐spined sticklebacks (Praebel et al., 2013; Schluter & Mcphail, 1992), two morphs of *S. thermalis* showed a high degree of similarity in the number and length of gill rakers, which might indicate that the morphological divergence of polymorphism displaying various traits and levels. Another possibility is that two morphs of *S. thermalis* may be on the way to differentiation with ongoing gene flow.

### 4.2 Deits

Our diet analysis revealed that two morphs of *S. thermalis* displayed low dietary overlap. The planktivorous morph predominately feeds on zooplankton and small fishes in the pelagic area, thus feeding partially on animals, while the benthivorous morph mainly feeds on periphytic algae and zoobenthos in lake benthic zones and the river, thus preferring a plant‐based diet. Differentiation of diets is a common phenomenon in schizothoracines. For example, two morphological form species of genus *Scizhothorax* co‐distribute within same river, but their diet compositions are divergence. Species with a sharp horny edge on the lower jaw often feed on appendiculate algae, whereas those without a sharp horny edge mainly feed on aquatic insects (He & Chen, 2006). The difference of trophic traits is primarily resulted from utilizing discrete food resource. Feeding on particular prey can result in plastic changes in trophic structure and may lead to greater morph specialization. Food type and quality may further change trophic morphologies. In an ecological context, the differentiation of trophic adaptive traits is important in resource partitioning and reducing intraspecific competition in a sympatric population by a decrease in dietary overlap (Smith & Skúlason, 1996; Swanson, Gibb, Marks, & Hendrickson, 2003).

### 4.3 Growth features

The growth of fishes can be affected by feed resource (quality and quantity), environmental conditions, inherent genetic factor, and physiological condition. Water on the Tibetan Plateau is characterized by lower water temperature, high concentration of dissolved oxygen, and oligotrophic conditions, which leads to slow growth, late sexual maturity, long life, and the production of fewer offspring of fishes at in higher altitude area in nature waters (Chen & Cao, 2000; Wang, 2016), and *S. thermalis* is no exception. Our results of the ARSS test indicated that there were significant differences in growth parameters (*L_∞_*, *k*, *φ*) between females and males in both the planktivorous morph and the benthivorous morph. Overall, in comparison to the benthivorous morph, the planktivorous had a higher growth performance index (*φ*) and a larger asymptotic value (*L_∞_*) at a lower growth coefficient (*k*). The difference in growth performance between two morphs is also found the lake charr (*S. namaycush*) in Rush Lake (Chavarie et al., 2016). Difference in growth characteristics between morphs might be relate to feeding biology (Hindar & Jonsson, 1982). The feeding strategy of species or morphs can affect the growth performance under similar environment conditions. For example, the piscivorous fish of *Oxygymnocypris stewartii* showed relatively higher growth performance than the co‐distributed omnivorous relatives (Jia & Chen, 2011). Jonsson and Jonsson (2001) also found that the somatic growth rate and maximum size of fish generally depended on the quality and quantity of food. Therefore, the individuals of planktivorous fish are much easier to obtain a high‐quality food of animal diets than those of benthivorous ones, which may facilitate growth (Jonsson & Jonsson, 1997, 1998).

### 4.4 Phylogenetic analyses

Resource polymorphism in fishes, such as salmonid species or cyprinid species, may be the result of phenotypic plasticity or trait heritability (Klemetsen, 2010; Seehausen & Wagner, 2014; Skúlason et al., 2019). In the African barb (*L. gananensis*), mouth polymorphism is attributed to phenotypic plasticity (Levin et al., 2019), while in the European whitefish (*C. lavaretus*), which exhibits differences in gill raker count, the trophic niches of littoral, pelagic, and profundal morphs are heritable (Praebel et al., 2013). Phylogenetic and AMOVA analyses did not support markedly genetic differentiation being two morphs of *S. thermalis*, rather confirmed that two morphs shared a more recent co‐ancestry and a common pool of genetic variation. Compared with sympatric species pairs, *G. e. eckloni* and *G. e. scoliostomus* in Lake Sunmcuo (Zhao et al., 2009), and *G. chui* and *G. scleracanthus* in Lake Langcuo (Chen et al., 2020), the *S. thermalis* exhibited a low level of genetic differentiation, even gene flow is almost unimpeded in our system. Our findings are similar to the lean and huronicus morphs (*S. namaycush*) in Rush Lake (Chavarie et al., 2016). Low levels of genetic differentiation between two morphs can be attributed to recent divergence and unsuitable genetic markers. In some of resource polymorphism species, morphs can be formed very quickly, even within one generation (Smith & Skúlason, 1996). The genetic relatedness between the two morphs is inferred from marker little influenced by selection in this study, but loci under divergent selection will be maintain stronger differentiation between the habitats than neutral loci (Feder & Nosil, 2010). Under both scenarios, maternal mitochondrial genes may be reflecting neutral population genetic processes (as opposed to selection reflect).

### 4.5 Ecological mechanisms of resource polymorphism in *S. thermalis*

Smith and Skúlason (1996) sketched a generalized possible steps and mechanisms leading to resource polymorphisms and speciation: (a) Ecological circumstances with a relaxation of interspecific completion and the availability of open niches appear essential for promoting resource polymorphisms; (b) divergent selection (e.g., selection against intermediates) and adaption to discrete resources increased phenotypic divergence of sympatric forms probably reduces competition; (c) a stable polymorphism with high gene flow; (d) divergent selection and reduced gene flow with reproductive isolation; this may result from prezygotic, such as spatial and temporal segregation in breeding, and ultimately leads to forming new species by ecological speciation. This model provides a suitable explanation of how to arise and maintain of resource polymorphism observed in *S. thermalis*.

Lake Amdo Tsonak Co is a high‐altitude headwater lake of the Salween River system on the Tibetan Plateau. Only few fish species resources for fish (*S. thermalis* and three species of *Triplophysa*) inhabit in the lake, but the lake has heterogeneous environment with more than two discrete habitats with distinctive food resources for fish. For example, the lake contains plentiful zooplankton in the pelagic area, abundant periphytic algae, and zoobenthos in the benthic zone, and a large amount of aquatic plants on its shoals. Thus, the availability of vacant niches and intense intraspecific competition might be fundamental prerequisites driving the differentiation in morphology. Ecological forms may establish barriers to gene flow, and reproductive isolation may occur when barriers are sufficient to prevent recent gene flow. During the field investigation in Lake Amdo Tsonak Co, we found two different types of spawning: river spawning and lacustrine spawning in northeast of the lake. Because planktivores inhabit only the lake and benthivores are distributed in the lake and its outlet and tributaries, we speculate that the planktivores spawn only on shoals within the lake, which are rich in aquatic plants, while the benthivores may spawn in watercourses. This difference in spawning ground indicates a possibility for the existence of partial spatial isolation. Hence, the morphological differentiation of *S. thermalis* might be further maintained via this partial spatial isolation.

## 5 CONCLUSION

Combining with morphological traits, growth features, and dietary preference, we confirmed that two morphs related to resource polymorphism, planktivorous, and benthivorous, in *S. thermalis* in Lake Amdo Tsonak Co. Two morphs display substantial difference in trophic characteristics (e.g., mouth position, jaw features, mucus pores, and pharyngeal teeth), feeding habit, and growth features. The pelagic–benthic resources partition and low species density in the Lake might have driven the initial morphological differentiation of *S. thermalis*. Also, the divergence in the two morphs might be ultimately maintained and reinforced by partial spatial isolation in the freshwater environment. However, our sampling was insufficient along the lake‐depth gradient (e.g., littoral, pelagic, and profundal) due to inconvenient traffic and extreme environmental conditions. Thus, the detailed distribution of the two morphs within lakes needs to be further investigated. It is also not clear whether the feeding difference between the two morphs maintains a long‐term or only a short‐term (seasonal variation) level; hence, a more reliable method that could reflect individual long‐term utilizing food resource (e.g., carbon and nitrogen stable isotope analysis) should be conducted to explore the dietary differences between the two morphs in future studies (Olson, Krabbenhoft, Hrabik, Mendsaikhan, & Jensen, 2019). In addition, the genetic basis underlying this process is unknown; thus, more sensitive genetic markers, such as single nucleotide polymorphism (SNP) markers, should be used to explore the differentiation process the two morphs. The high‐altitude freshwater lakes in the Qinghai‐Tibetan Plateau commonly hold diverse environmental conditions, where interspecific competitors are few and available resources are heterogeneous. Thus, resource polymorphism might largely underestimate in the Plateau lakes (Chen et al., 2020; Zhao et al., 2009). In this respect, resource polymorphism in these natural systems provides unique opportunities for studying the process of speciation.

## CONFLICT OF INTEREST

The authors declare that we have no competing interests related to this work.

## AUTHOR CONTRIBUTION

**Jialing Qiao:** Data curation (lead); Methodology (equal); Software (equal); Writing‐original draft (lead); Writing‐review & editing (lead). **Jiaxin Hu:** Data curation (equal); Methodology (equal); Software (equal). **Qin Xia:** Writing‐review & editing (lead). **Ren Zhu:** Investigation (lead); Methodology (equal). **Kang Chen:** Data curation (supporting); Methodology (equal); Software (supporting). **Jie Zhao:** Methodology (equal); Software (equal). **Yunzhi Yan:** Methodology (supporting); Software (supporting). **Ling Chu:** Conceptualization (lead); Resources (lead). **Dekui He:** Conceptualization (lead); Investigation (lead); Resources (lead); Writing‐review & editing (lead).

## Supporting information

Fig S1

Fig S2

Fig S3

Table S1

## Data Availability

All raw data for morphometric, dietary, growth and phylogenetic analyses, and R code necessary for recreating the analyses in this paper are deposited to Dryad at the following URL: https://doi.org/10.5061/dryad.nvx0k6dnt
