# Supplementary figures and images for: Pelagic–benthic resource polymorphism in Schizopygopsis thermalis Herzenstein 1891 (Pisces, Cyprinidae) in a headwater lake in the Salween River system on the Tibetan Plateau
Source: Ecol Evol. 2020 Jul 8;10(14):7431–44. doi: 10.1002/ece3.6470 (PMC7391544; doi:10.1002/ece3.6470)

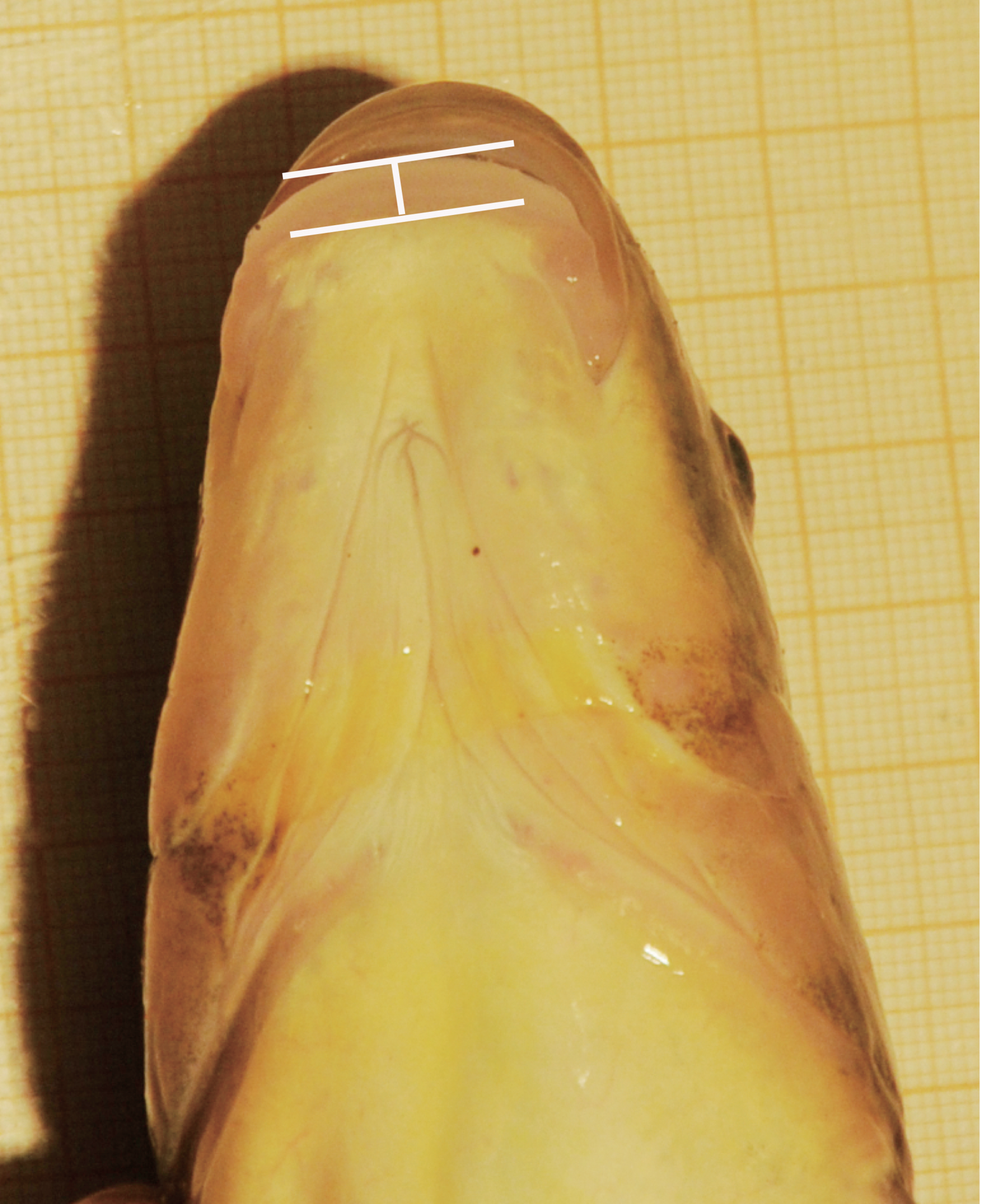

Supplement: Supplementary file 1 — Fig S1 [file ECE3-10-7431-s001.pdf]

(a)

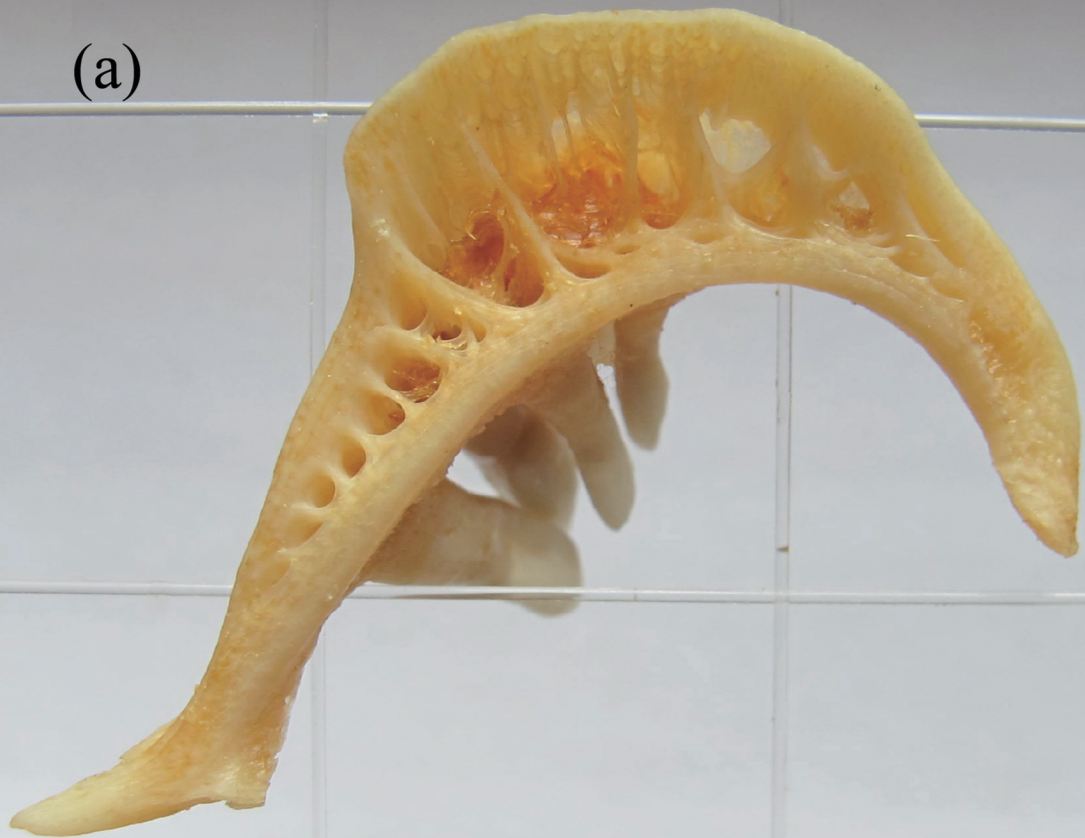

(b)

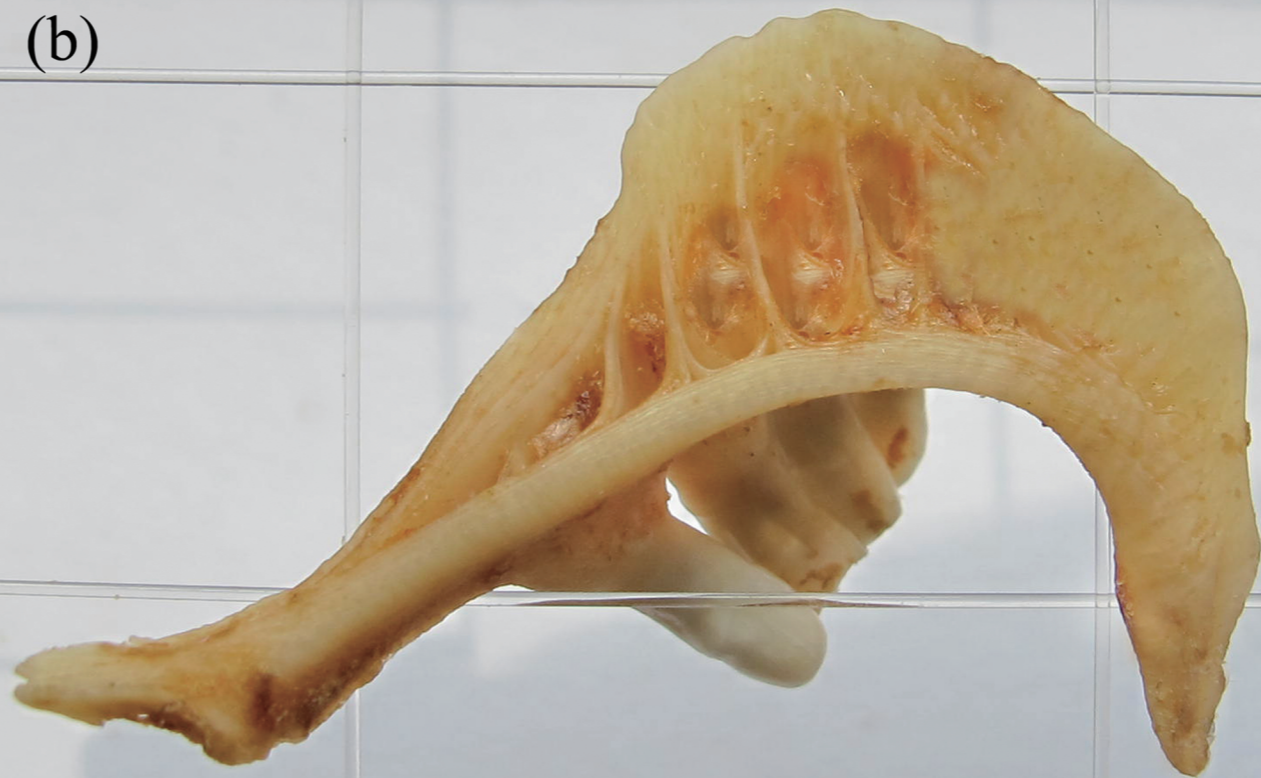

Supplement: Supplementary file 2 — Fig S2 [file ECE3-10-7431-s002.pdf]

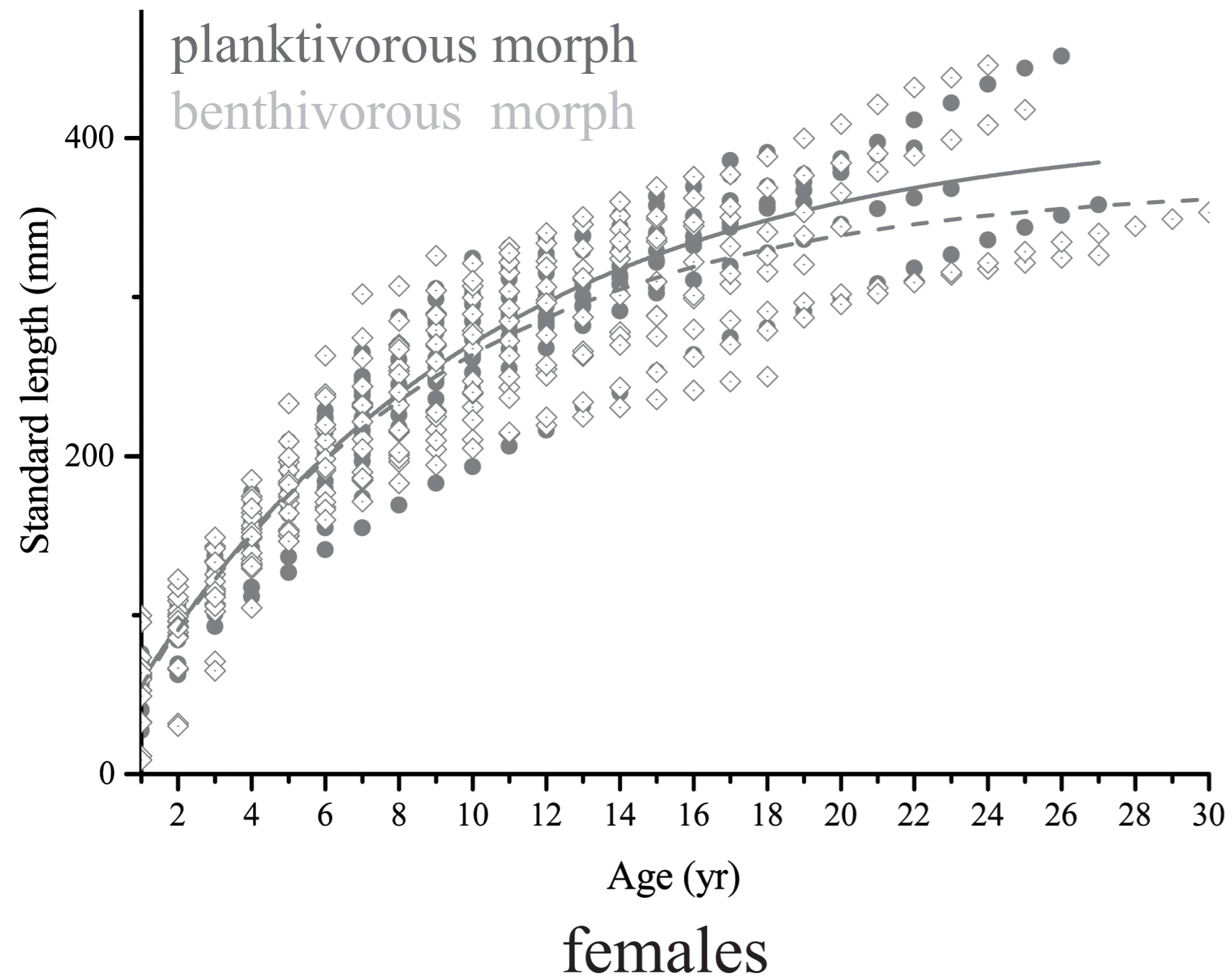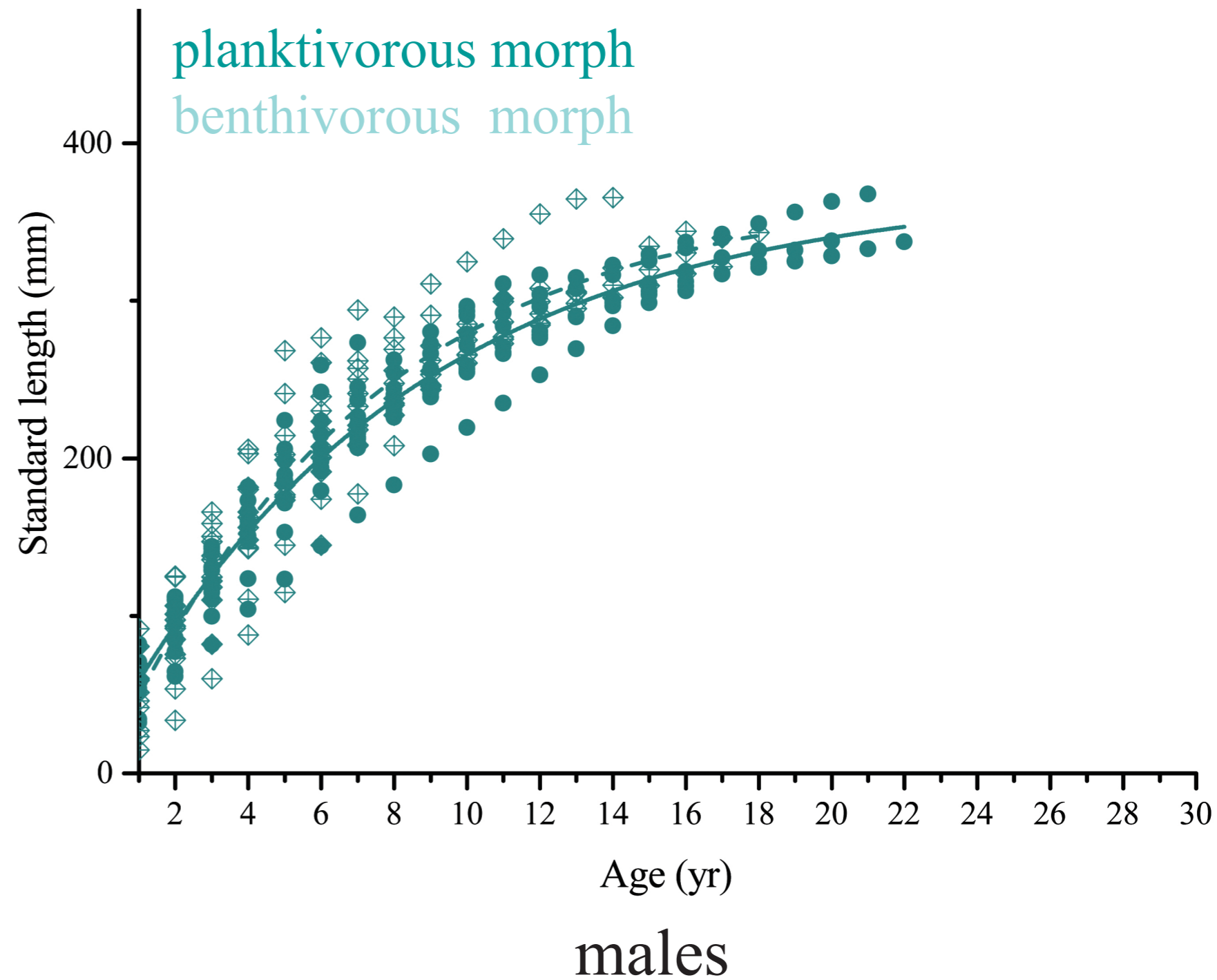

Supplement: Supplementary file 3 — Fig S3 [file ECE3-10-7431-s003.pdf]
