# Supplementary material for: Pelagic–benthic resource polymorphism in Schizopygopsis thermalis Herzenstein 1891 (Pisces, Cyprinidae) in a headwater lake in the Salween River system on the Tibetan Plateau
Source: Ecol Evol. 2020 Jul 8;10(14):7431–44. doi: 10.1002/ece3.6470 (PMC7391544; doi:10.1002/ece3.6470)
Supplement: Supplementary file 4 — Table S1 [file ECE3-10-7431-s004.docx]

**Appendices**

**Table S1.** Primer sequences used in this study.

| Gene | Primer name | Sequence (5’-3’) | Product length(bp) | primer extension(t) | Reference |
| --- | --- | --- | --- | --- | --- |
| Cyt *b* | L14724 | GACTTGAAAAACCACCGTTG | 1140 | 90 | He and Chen (2006) |
|  | H15915 | CTCCGATCTCCGGATTACAAGAC |  |  |  |
| D-loop | GEDL200 | ACCCCTGGCTCCCAAAGC | 839 | 45 | Liang et al. (2017) |

He, D. & Chen, Y. (2006). Biogeography and molecular phylogeny of the genus *Schizothorax* (Teleostei: Cyprinidae) in China inferred from cytochrome b sequences. *Journal of Biogeography*, 33, 1448-1460. [https://doi.org/10.1111/j.1365-2699.2006.01510.x](https://xs.scihub.ltd/https://doi.org/10.1111/j.1365-2699.2006.01510.x)

Liang, Y., He, D., Jia, Y., Sun, H. & Chen, Y. (2017). Phylogeographic studies of schizothoracine fishes on the central Qinghai-Tibet Plateau reveal the highest known glacial microrefugia. *Scientific reports*, 7, 10983. [https://xs.scihub.ltd/https://doi.org/10.1038/s41598-017-11198-w](https://xs.scihub.ltd/https:/doi.org/10.1038/s41598-017-11198-w)

Figure S1. The method used to measure the width of the horny edge of the lower jaw at the widest part.

Figure S2. Two different types of pharyngeal teeth. Two rows of pharyngeal teeth are shown in (a), while a single row of pharyngeal teeth is exhibited in (b).

Figure S3. Relationship between age and the back-calculated standard length for females, males of the planktivorous morph and benthivorous morph.
